# Trajectories of grip strength decline and risk of new-onset cardiovascular disease: evidence from the HRS and ELSA cohorts

**DOI:** 10.3389/fpubh.2026.1836439

**Published:** 2026-06-26

**Authors:** Yong Mo, Yiyi Zhang, Changcheng Li, Wanying Zhang, Ping Chen, Linjiang Song, Yulin Li

**Affiliations:** 1 School of Medical and Life Sciences, Chengdu University of Traditional Chinese Medicine, Chengdu, Sichuan, China; 2 Hospital of Chengdu University of Traditional Chinese Medicine, Chengdu University of Traditional Chinese Medicine, Chengdu, Sichuan, China; 3 Translational Chinese Medicine Key Laboratory of Sichuan Province, Sichuan Academy of Chinese Medicine Sciences, Sichuan Institute for Translational Chinese Medicine, Chengdu, Sichuan, China; 4 Chengdu Fifth People’s Hospital (The Second Clinical Medical College, Affiliated Fifth People’s Hospital of Chengdu University of Traditional Chinese Medicine), Chengdu, Sichuan, China

**Keywords:** aging, cardiovascular disease, grip strength, grip strength decline, longitudinal cohort, trajectory analysis

## Abstract

**Background:**

Handgrip strength is an important indicator of overall health, yet most studies rely on single measurements. Evidence on its long-term trajectories and new-onset cardiovascular disease (CVD) risk remains limited. This study examined associations of baseline handgrip strength and its trajectories with new-onset CVD in two aging cohorts.

**Methods:**

Data were from the Health and Retirement Study (HRS) in the United States and the English Longitudinal Study of Ageing (ELSA). Participants aged ≥50 years without baseline CVD were included. Handgrip strength across three waves was used to construct trajectories via group-based trajectory modeling. Cox regression assessed associations with new-onset CVD. Fine–Gray models, discrete-time logistic regression, restricted cubic splines, and mediation analysis (depression) were conducted.

**Results:**

A total of 4,984 (HRS) and 4,982 (ELSA) participants were included. Over median follow-ups of 10 and 12 years, 1,438 and 1,184 CVD events occurred, respectively. Higher baseline handgrip strength was associated with lower CVD risk (HRS: HR = 0.79, 95% CI: 0.73–0.86; ELSA: HR = 0.83, 95% CI: 0.76–0.91). Two trajectories were identified: accelerated decline and steady decline. Compared with steady decline, accelerated decline was linked to higher CVD risk (HRS: HR = 1.44, 95% CI: 1.22–1.70; ELSA: HR = 1.31, 95% CI: 1.08–1.58). Results were consistent across sensitivity analyses. A linear inverse association was observed, and depression partially mediated this relationship.

**Conclusion:**

Lower baseline handgrip strength and accelerated decline are associated with increased CVD risk. Longitudinal monitoring may aid early risk identification and prevention.

## 1 Introduction

Cardiovascular disease (CVD) remains one of the leading causes of morbidity and mortality among older adults worldwide (1, 2). As global population aging accelerates, the absolute burden of CVD is expected to rise substantially. This trend highlights the urgent need for simple and cost-effective indicators that enable early identification of high-risk individuals and facilitate timely preventive interventions (3). In this context, declining physical function has emerged as a key phenotype of biological aging. Progressive deterioration in muscle function has been strongly associated with increased risks of chronic disease, functional impairment, and mortality (4, 5).

Handgrip strength, typically measured using a handheld dynamometer, is a simple, objective, reproducible, and noninvasive indicator of muscle function. It has been widely used in epidemiological studies of aging populations (6). Increasing evidence suggests that handgrip strength is an important biomarker of systemic health. It reflects the integrated effects of neuromuscular, metabolic, and inflammatory pathways and is often used as a practical surrogate marker of frailty and biological aging (7, 8).

Accumulating evidence consistently demonstrates an inverse association between handgrip strength and adverse cardiovascular outcomes. Even after adjustment for traditional risk factors such as age, sex, body mass index, and comorbidities, lower baseline handgrip strength remains independently associated with increased risks of cardiovascular disease events, cardiovascular mortality, and all-cause mortality (9–11). These findings support the notion that handgrip strength may serve as an indicator of frailty or accelerated biological aging. Meta-analyses have further confirmed a significant dose–response relationship across diverse populations (12).

However, most previous studies have relied on a single baseline measurement of handgrip strength and treated it as a static exposure. Such an approach cannot capture the dynamic decline in muscle function that occurs during the aging process (13). Emerging evidence suggests that longitudinal changes in physiological indicators may provide more sensitive markers of biological aging and offer improved prognostic information compared with single measurements (14).

Despite these advances, several important knowledge gaps remain. Studies examining the relationship between dynamic changes in handgrip strength—particularly the rate of decline or heterogeneous trajectory patterns—and subsequent cardiovascular disease risk are still limited (15). Muscle function trajectories during aging show considerable inter-individual heterogeneity, ranging from stable maintenance at high levels to gradual decline or accelerated loss. Whether these distinct patterns differentially predict cardiovascular disease incidence remains insufficiently explored in longitudinal studies (16). In addition, most existing research has been conducted within single-country cohorts, which limits the generalizability and reproducibility of the findings across different populations and healthcare systems (17).

To address these gaps, the present study utilized two nationally representative longitudinal aging cohorts: the Health and Retirement Study (HRS) in the United States and the English Longitudinal Study of Ageing (ELSA) in the United Kingdom. Using repeated assessments of handgrip strength, we examined dynamic changes in muscle strength from two complementary perspectives: baseline handgrip strength and trajectory patterns identified using latent trajectory modeling. We then prospectively investigated the associations between these dynamic indicators and the risk of new-onset CVD. By determining whether handgrip strength trajectories can serve as early functional markers of cardiovascular risk, this study aims to provide robust and reproducible epidemiological evidence to inform prevention strategies and risk stratification in rapidly aging populations (18, 19).

## 2 Materials and methods

### 2.1 Study population

The ELSA, led by a consortium that includes University College London and the National Centre for Social Research, is a nationally representative cohort study designed to monitor the health, economic conditions, and social circumstances of adults aged ≥ 50 years residing in private households in England. The HRS, conducted by the University of Michigan with support from the National Institute on Aging, is a nationally representative longitudinal survey of adults aged ≥ 51 years from the non-institutionalized civilian population of the United States. The study examines the dynamic interplay among health, economic, and social factors in later life.

**Figure 1:**
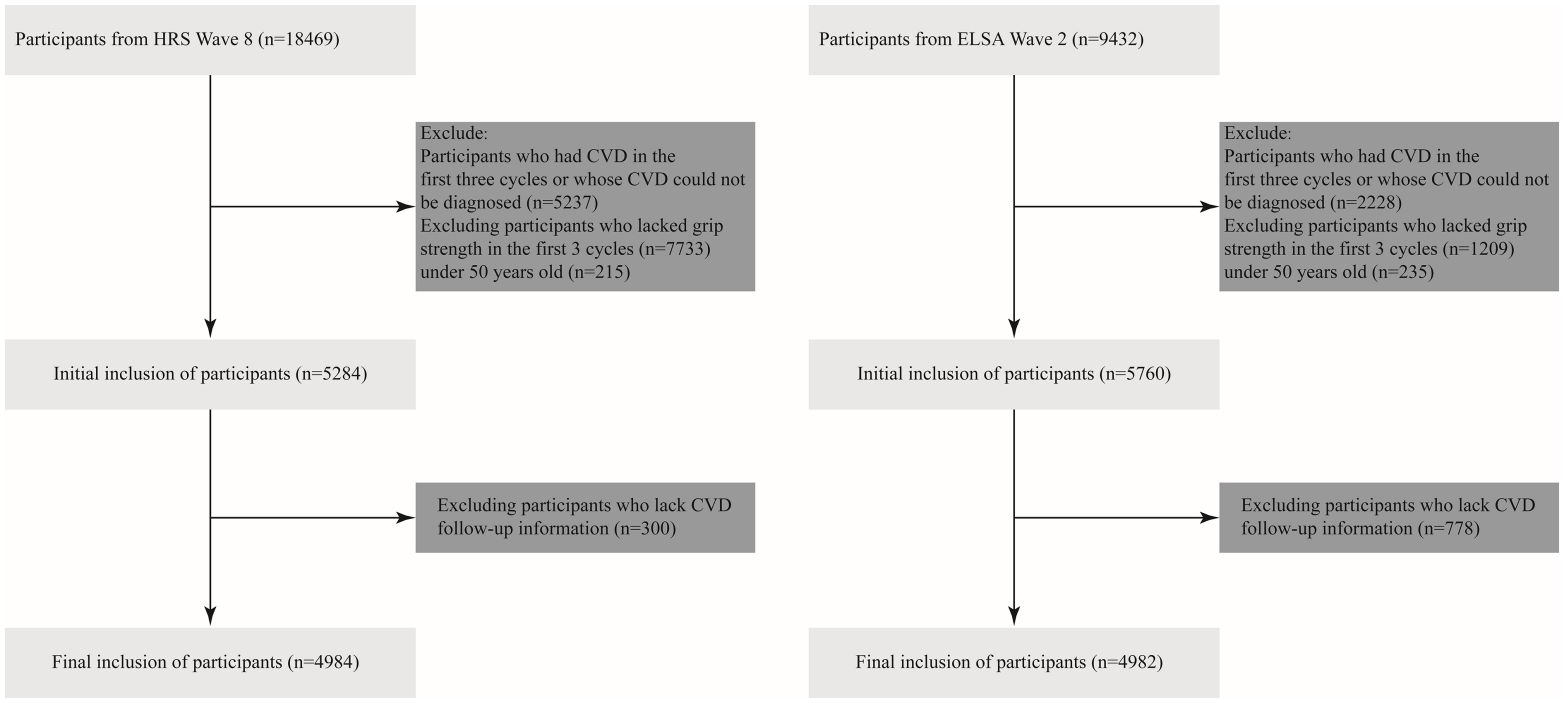
Flowchart of participant selection.

The present investigation employed a longitudinal cohort design using data from HRS and ELSA. Both cohorts are ongoing, nationally representative studies of community-dwelling older adults. Standardized interviews and physical examinations are conducted biennially to collect detailed information on sociodemographic characteristics, health status, functional capacity, and health-related behaviors. A total of 18,469 participants were initially identified in the HRS database. After applying predefined exclusion criteria, 13,485 participants were excluded. Specifically, participants were excluded if they had cardiovascular disease (CVD) or lacked diagnostic information on CVD during the first three waves (*n* = 5,237), had missing handgrip strength measurements during the first three waves (*n* = 7,733), were younger than 50 years of age (*n* = 215), or lacked follow-up information on CVD (*n* = 300). After these exclusions, 4,984 participants from the HRS database were included in the final analysis (Figure 1). In the ELSA database, 9,432 participants were initially identified. Among them, 4,450 participants were excluded according to the predefined criteria. Exclusions included participants who had CVD or lacked diagnostic information on CVD during the first three waves (*n* = 2,228), those with missing handgrip strength measurements during the first three waves (*n* = 1,209), individuals younger than 50 years of age (*n* = 235), and participants without follow-up information on CVD (*n* = 778). As a result, 4,982 participants from the ELSA database were included in the final analysis (Figure 1).

To minimize potential reverse causation, participants with prevalent CVD during the first three survey waves were excluded prior to trajectory modeling. Handgrip strength trajectories were constructed using repeated measurements obtained during the first three waves, and follow-up for new-onset CVD began after trajectory classification was completed.

### 2.2 Assessment of grip strength and construction of grip strength trajectories

Data from HRS and ELSA were harmonized for analysis. Both cohorts conduct biennial follow-up assessments and collect health-related data through standardized interviews and objective physical measurements. Grip strength was assessed by trained personnel according to a standardized protocol. Participants were typically seated and instructed to perform repeated maximal efforts using a handheld dynamometer, with measurements obtained for both hands. To reduce random measurement error, the highest value recorded from either hand within each wave was used to represent grip strength for that assessment cycle. To ensure cross-cohort comparability, standardized grip strength variables from the publicly available datasets were used. Implausible values were reviewed for validity. Only participants with valid baseline grip strength measurements were included in subsequent analyses.

To characterize long-term changes in muscle strength, grip strength trajectories were constructed. Consistent with prior trajectory analyses, data from three consecutive interview waves were used to model longitudinal patterns. Participants were required to have valid grip strength measurements in all three waves. Time was defined as years since baseline, with approximately two-year intervals between waves. Group-Based Trajectory Modeling (GBTM), based on finite mixture modeling, was applied to identify latent subgroups with distinct grip strength trajectories. Time was specified as the independent variable and grip strength as the dependent variable. Models with varying numbers of latent groups were estimated. The optimal number of trajectory groups was determined according to predefined statistical criteria. Each participant was subsequently assigned to a trajectory group based on the maximum posterior probability. Trajectory membership was treated as a categorical exposure in subsequent analyses examining associations with new-onset CVD.

### 2.3 Assessment of incident cardiovascular disease

CVD was defined as the first self-reported physician diagnosis of cardiovascular conditions during follow-up, including heart disease, myocardial infarction, angina, congestive heart failure, stroke, and other physician-diagnosed cardiovascular events reported in the original questionnaires. In both cohorts, diagnostic information was collected at each wave using standardized questionnaires. To preserve the temporal sequence between exposure and outcome, all participants were required to be free of CVD during the trajectory modeling phase (the first three waves). Individuals with prevalent CVD during the first three waves were excluded. New-onset CVD was defined as the wave in which CVD was first reported. Follow-up for CVD began in the wave immediately after completion of trajectory modeling and continued until the occurrence of CVD, loss to follow-up, or the end of follow-up. This design ensured that grip strength trajectories preceded the outcome and minimized the potential for reverse causation.

### 2.4 Covariates

Sociodemographic and lifestyle variables were included as covariates. These included Age, Sex, Race, Education, Wealth, BMI, Drinking status, Smoking status, diabetes, cancer, and Physical activity. Smoking status was categorized as never smoker or current smoker. Drinking status was classified as non-drinker or current drinker. BMI was calculated as weight (kg) divided by height (m) squared. Diabetes was defined by an affirmative response to the question, “Doctor told you have diabetes.”

### 2.5 Statistical analyses

Before conducting statistical analyses, the distribution of continuous variables was evaluated using the Shapiro–Wilk test together with visual inspection of histograms and density plots. Most continuous variables showed non-normal distributions. Therefore, continuous variables are presented as median (interquartile range, IQR), and between-group comparisons were performed using the Mann–Whitney U test. Categorical variables are presented as counts and percentages, and comparisons were conducted using the chi-square test (Supplementary Figure S1). GBTM was used to identify distinct longitudinal patterns of grip strength. This method applies maximum likelihood estimation within a finite mixture modeling framework to classify individuals with similar developmental trajectories into latent subgroups. Trajectory modeling was conducted separately for each cohort. Grip strength data from the first three waves were included, and time was defined as years since baseline. Models specifying one to three trajectory groups with linear and quadratic polynomial terms were estimated. Model selection was based on the Bayesian Information Criterion (BIC), Akaike Information Criterion (AIC), log-likelihood values, mean posterior probability, and clinical interpretability. Additional selection criteria included a minimum trajectory group size >5% and a mean posterior probability ≥0.70. Participants were assigned to trajectory groups according to the maximum posterior probability principle. The final two-trajectory model was selected because it provided the optimal balance between model fit, classification quality, trajectory stability, and interpretability. Associations between grip strength, grip strength trajectories, and new-onset CVD were evaluated using multivariable Cox proportional hazards regression models. Time-to-event was defined as the interval from completion of the initial three-wave assessment to the first occurrence of CVD, censoring, or the end of follow-up. Hazard ratios (HR) and 95% confidence intervals (CI) were calculated. Model 1 was unadjusted. Model 2 adjusted for Age, Sex, Race, Education, Wealth, and BMI. Model 3 further adjusted for Drinking status, Smoking status, and diabetes. Model 4 additionally adjusted for cancer, and Physical activity. The proportional hazards assumption was assessed using Schoenfeld residuals, and no substantial violations were detected. To account for the possibility that death could preclude the occurrence of CVD, competing risk regression was performed using the Fine–Gray subdistribution hazard model, with death treated as a competing event. Subdistribution hazard ratios (sHR) and 95% CI were reported to evaluate the robustness of the findings within a competing risk framework. As a sensitivity analysis, discrete-time logistic regression models were fitted using follow-up wave as the time unit. The dataset was restructured into a person-period format, with each participant contributing one observation per wave until new-onset CVD or censoring. Restricted cubic spline (RCS) analysis was conducted to assess potential nonlinear associations between grip strength and new-onset CVD risk. Mediation analyses were conducted to explore whether depression mediated the association between baseline handgrip strength and new-onset CVD risk. Baseline handgrip strength was treated as the exposure, depression as the potential mediator, and new-onset CVD as the outcome. Depression was assessed prior to incident CVD during follow-up to preserve the temporal sequence among exposure, mediator, and outcome. Mediation effects were estimated after adjustment for potential confounding variables, and the proportion mediated was subsequently calculated. All statistical analyses were performed using IBM SPSS Statistics (version 24.0) and R software (version 4.3.0). A two-sided *p* value < 0.05 was considered statistically significant.

## 3 Results

### 3.1 Baseline characteristics of participants according to new-onset CVD status

Tables 1, 2 summarize baseline characteristics of participants with and without new-onset CVD in the HRS and ELSA cohorts. A total of 4,984 participants from the HRS cohort who were free of CVD during the first three waves were included. The median age was 66.00 years. Among them, 3,031 participants were female (60.81%) and 1,953 were male (39.19%). During a median follow-up period of 10 years, 1,438 participants developed new-onset CVD. The median age of these individuals was 68.00 years.

**Table 1:** Baseline characteristics of participants stratified by new-onset cardiovascular disease (CVD) in the HRS cohort.

Characteristic	*N*	Overall *N* = 4,984	No New-onset CVD *N* = 3,546	New-onset CVD *N* = 1,438	*p*-value
Sex^b^, *n* (%)	4,984				0.068
Female		3,031 (60.81%)	2,185 (61.62%)	846 (58.83%)	
Male		1,953 (39.19%)	1,361 (38.38%)	592 (41.17%)	
Age(year)^a^, M (Q_1_, Q_3_)	4,984	66.00 (58.00–73.00)	65.00 (57.00–72.00)	68.00 (62.00–75.00)	<0.001
Race^b^, *n* (%)	4,984				0.065
Non-hispanic white		3,811 (76.46%)	2,681 (75.61%)	1,130 (78.58%)	
Non-hispanic blacks		642 (12.88%)	462 (13.03%)	180 (12.52%)	
Hispanic		430 (8.63%)	326 (9.19%)	104 (7.23%)	
Other race		101 (2.03%)	77 (2.17%)	24 (1.67%)	
Education^b^, *n* (%)	4,984				0.002
High school or less		948 (19.02%)	652 (18.39%)	296 (20.58%)	
Some college		2,814 (56.46%)	1,978 (55.78%)	836 (58.14%)	
College graduate or above		1,222 (24.52%)	916 (25.83%)	306 (21.28%)	
Wealth^a^, M (Q_1_, Q_3_)	4,984	253,500.00 (72,955.59—602,250.00)	262,750.00 (77,031.00—612,500.00)	230,100.00 (64,200.00—569,000.00)	0.048
BMI^a^, M (Q_1_, Q_3_)	4,984	27.30 (24.30–30.90)	27.30 (24.10–30.70)	27.50 (24.80–31.50)	<0.001
Hypertension^b^, *n* (%)	4,984				<0.001
No		2,593 (52.03%)	1,984 (55.95%)	609 (42.35%)	
Yes		2,391 (47.97%)	1,562 (44.05%)	829 (57.65%)	
Diabetes ^b^, *n* (%)	4,984				<0.001
No		4,218 (84.63%)	3,074 (86.69%)	1,144 (79.55%)	
Yes		766 (15.37%)	472 (13.31%)	294 (20.45%)	
Smoking status^b^, *n* (%)	4,984				0.596
No		2,279 (45.73%)	1,613 (45.49%)	666 (46.31%)	
Yes		2,705 (54.27%)	1,933 (54.51%)	772 (53.69%)	
Drinking status^b^, *n* (%)	4,984				0.009
No		2,272 (45.59%)	1,575 (44.42%)	697 (48.47%)	
Yes		2,712 (54.41%)	1,971 (55.58%)	741 (51.53%)	
Physical activity ^b^, *n* (%)	4,984				0.105
No		1,097 (22.01%)	759 (21.40%)	338 (23.50%)	
Yes		3,887 (77.99%)	2,787 (78.60%)	1,100 (76.50%)	
Cancer^b^, *n* (%)	4,984				0.156
No		4,387 (88.02%)	3,136 (88.44%)	1,251 (87.00%)	
Yes		597 (11.98%)	410 (11.56%)	187 (13.00%)	
CSE-D ^a^, M (Q_1_, Q_3_)	4,984	1.00 (0.00–2.00)	0.00 (0.00–2.00)	1.00 (0.00–2.00)	0.001
Grip strength ^a^, M (Q_1_, Q_3_)	4,984	30.00 (24.50–40.50)	30.50 (25.00–40.50)	29.50 (23.00–40.00)	0.002
Grip trajectory^b^, *n* (%)	4,984				0.072
Accelerated decline		1,495 (30.00%)	1,090 (30.74%)	405 (28.16%)	
Steady decline		3,489 (70.00%)	2,456 (69.26%)	1,033 (71.84%)	

a, Kruskal-Wallis rank sum test; b, Chi-square test; SD, standard deviation.

**Table 2:** Baseline characteristics of participants stratified by new-onset cardiovascular disease (CVD) in the ELSA cohort.

Characteristic	N	Overall *N* = 4,982	No New-onset CVD *N* = 3,798	New-onset CVD *N* = 1,184	*p*-value
Sex^b^, *n* (%)	4,982				0.008
Female		2,816 (56.52%)	2,186 (57.56%)	630 (53.21%)	
Male		2,166 (43.48%)	1,612 (42.44%)	554 (46.79%)	
Age (year) ^a^, M (Q_1_, Q_3_)	4,982	63.00 (57.00–71.00)	62.00 (57.00–70.00)	67.00 (60.00–74.00)	<0.001
Race^b^, n (%)	4,982				0.228
No white		73 (1.47%)	60 (1.58%)	13 (1.10%)	
White		4,909 (98.53%)	3,738 (98.42%)	1,171 (98.90%)	
Education^b^, *n* (%)	4,982				0.219
Before high school		2,332 (46.81%)	1,748 (46.02%)	584 (49.32%)	
High school		950 (19.07%)	728 (19.17%)	222 (18.75%)	
Junior college		999 (20.05%)	775 (20.41%)	224 (18.92%)	
College graduate or above		701 (14.07%)	547 (14.40%)	154 (13.01%)	
Wealth ^a^, M (Q_1_, Q_3_)	4,982	210,000.00 (120,050.00—346,000.00)	210,000.00 (123,700.00—352,000.00)	205,400.00 (108,540.00—328,000.00)	0.019
BMI ^a^, M (Q_1_, Q_3_)	4,982	27.18 (24.63–30.52)	27.11 (24.52–30.35)	27.65 (25.05–31.08)	<0.001
Hypertension ^b^, *n* (%)	4,982				<0.001
No		3,185 (63.93%)	2,531 (66.64%)	654 (55.24%)	
Yes		1,797 (36.07%)	1,267 (33.36%)	530 (44.76%)	
Diabetes ^b^, *n* (%)	4,982				0.266
No		4,675 (93.84%)	3,572 (94.05%)	1,103 (93.16%)	
Yes		307 (6.16%)	226 (5.95%)	81 (6.84%)	
Smoking status^b^, *n* (%)	4,982				0.071
No		1,950 (39.14%)	1,513 (39.84%)	437 (36.91%)	
Yes		3,032 (60.86%)	2,285 (60.16%)	747 (63.09%)	
Drinking status^b^, *n* (%)	4,982				0.236
No		385 (7.73%)	284 (7.48%)	101 (8.53%)	
Yes		4,597 (92.27%)	3,514 (92.52%)	1,083 (91.47%)	
Physical activity ^b^, *n* (%)	4,982				0.010
No		909 (18.25%)	663 (17.46%)	246 (20.78%)	
Yes		4,073 (81.75%)	3,135 (82.54%)	938 (79.22%)	
Cancer^b^, *n* (%)	4,982				0.913
No		4,653 (93.40%)	3,548 (93.42%)	1,105 (93.33%)	
Yes		329 (6.60%)	250 (6.58%)	79 (6.67%)	
CSE-D ^a^, M (Q_1_, Q_3_)	4,982	1.00 (0.00–2.00)	1.00 (0.00–2.00)	1.00 (0.00–2.00)	0.002
Grip strength ^a^, M (Q_1_, Q_3_)	4,982	56.00 (44.00–77.00)	56.00 (45.00–77.00)	56.00 (43.00–77.00)	0.045
Grip trajectory ^b^, *n* (%)	4,982				0.713
Accelerated decline		1,808 (36.29%)	1,373 (36.15%)	435 (36.74%)	
Steady decline		3,174 (63.71%)	2,425 (63.85%)	749 (63.26%)	

a, Kruskal-Wallis rank sum test; b, Chi-square test; SD, standard deviation.

Compared with participants who remained free of CVD, those who developed CVD were older (median 68.00 vs. 65.00, *p* < 0.001), had lower household income (median 230,100.00 vs. 262,750.00, *p* = 0.048), and had higher BMI (median 27.50 vs. 27.30, *p* < 0.001). A greater proportion of alcohol consumption was also observed among participants who developed CVD (51.53% vs. 48.47%, *p* = 0.009). In addition, individuals who developed CVD had lower median handgrip strength compared with those who did not (median 29.50 vs. 30.50, *p* = 0.002). The distribution of participants categorized as having high handgrip strength also differed between groups (57.65% vs. 42.35%, *p* < 0.001) (Table 1).

In the ELSA cohort, 4,982 participants who were free of CVD during the first three waves were included in the analysis. The median age was 63.00 years. Among them, 2,816 participants were female (56.52%) and 2,166 were male (43.48%). During a median follow-up of 12 years, 1,184 participants developed new-onset CVD, with a median age of 67.00 years.

Compared with participants without new-onset CVD, those who developed CVD were older (median 67.00 vs. 62.00, *p* < 0.001), had lower household income (median 205,400.00 vs. 210,000.00, *p* = 0.019), and had higher BMI (median 27.65 vs. 27.11, *p* < 0.001) (Table 2).

### 3.2 Associations between handgrip strength, its trajectories, and the risk of new-onset CVD

Table 3 presents the associations between baseline handgrip strength, its trajectories, and new-onset CVD risk in the two cohorts using Cox proportional hazards regression models. In the crude model, baseline handgrip strength was inversely associated with the risk of new-onset CVD (HRS: HR = 0.89, 95% CI: 0.84–0.94, *p* < 0.001; ELSA: HR = 0.90, 95% CI: 0.84–0.95, *p* < 0.001).

**Table 3:** Association between grip strength and its variation trajectory and the risk of new-onset CVD.

Exposure Variable	Model 1		Model 2		Model 3		Model 4	
	HR (95% CI)	*p*-value	HR (95% CI)	*p*-value	HR (95% CI)	*p*-value	HR (95% CI)	*p*-value
HRS								
Grip per 1 SD	0.89 (0.84 ~ 0.94)	<0.001***	0.76 (0.70 ~ 0.82)	<0.001***	0.79 (0.72 ~ 0.86)	<0.001***	0.79 (0.73 ~ 0.86)	<0.001***
Grip trajectory								
Steady decline	1.00 (Reference)	—	1.00 (Reference)	—	1.00 (Reference)	—	1.00 (Reference)	—
Accelerated decline	1.18 (1.05 ~ 1.32)	0.005**	1.52 (1.28 ~ 1.79)	<0.001***	1.46 (1.24 ~ 1.73)	<0.001***	1.44 (1.22 ~ 1.70)	<0.001***
P for trend	0.91 (0.85 ~ 0.97)	0.005**	0.78 (0.71 ~ 0.86)	<0.001***	0.80 (0.72 ~ 0.88)	<0.001***	0.81 (0.73 ~ 0.89)	<0.001***
ELSA								
Grip per 1 SD	0.90 (0.84 ~ 0.95)	<0.001***	0.81 (0.74 ~ 0.89)	<0.001***	0.82 (0.75 ~ 0.90)	<0.001***	0.83 (0.76 ~ 0.91)	<0.001***
Grip trajectory								
Steady decline	1.00 (Reference)	—	1.00 (Reference)	—	1.00 (Reference)	—	1.00 (Reference)	—
Accelerated decline	1.09 (0.97 ~ 1.22)	0.165	1.33 (1.11 ~ 1.61)	0.002**	1.33 (1.10 ~ 1.60)	0.003**	1.31 (1.08 ~ 1.58)	0.005**
P for trend	1.00 (0.99 ~ 1.00)	0.165	0.99 (0.99 ~ 1.00)	0.002**	0.99 (0.99 ~ 1.00)	0.003**	0.99 (0.99 ~ 1.00)	0.005**

Model 1: crude.

Model 2: adjust: Age, Sex, Race, Education, marital status, Wealth, BMI.

Model 3: adjust: Age, Sex, Race, Education, marital status, Wealth, BMI, Hypertension, Diabetes, Drinking status, Smoking status.

Model 4: adjust: Age, Sex, Race, Education, marital status, Wealth, BMI, Hypertension, Diabetes, Drinking status, Smoking status, Physical activity, Cancer.

Significance: *p < 0.05, **p < 0.01, ***p < 0.001.

After adjustment for potential confounders in Model 4—including age, sex, race, education, wealth, BMI, drinking status, smoking status, hypertension, diabetes, physical activity, and cancer—the inverse association remained stable in both cohorts (HRS: adjusted HR = 0.79, 95% CI: 0.73–0.86, *p* < 0.001; ELSA: adjusted HR = 0.83, 95% CI: 0.76–0.91, *p* < 0.001). Specifically, each one–standard deviation increase in handgrip strength was associated with a 17–21% reduction in the risk of new-onset CVD across both cohorts.

**Figure 2:**
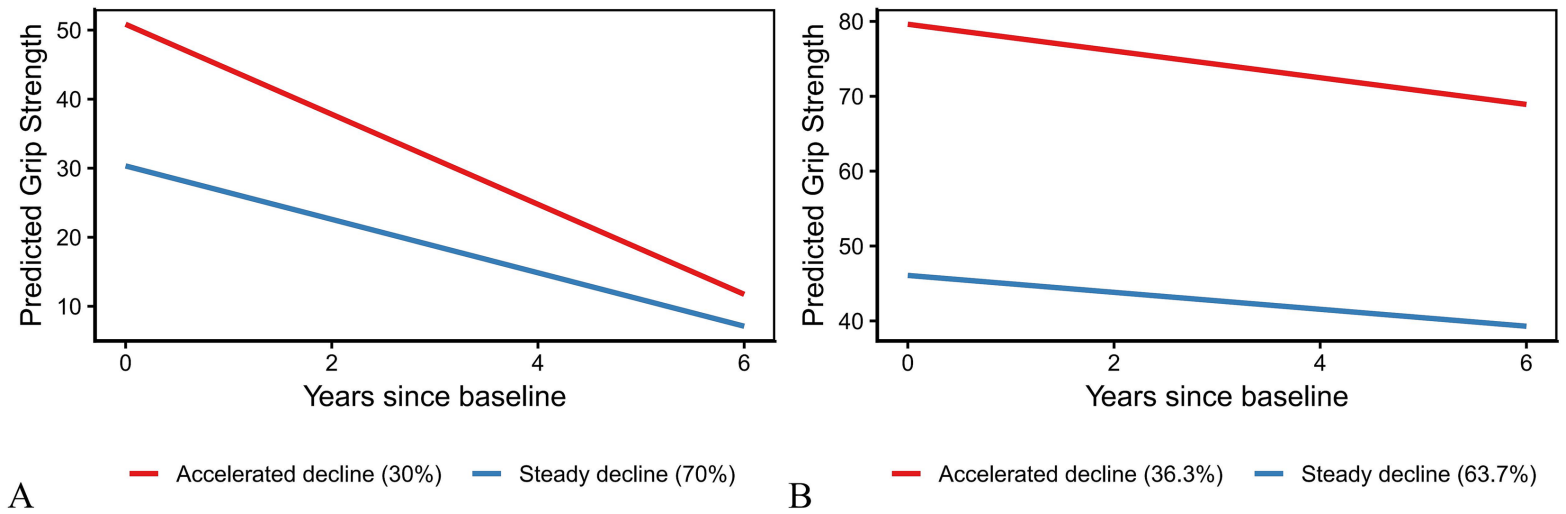
Grip strength trajectories identified using trajectory analysis in the study population: **(A)** HRS cohort; **(B)** ELSA cohort.

GBTM identified two distinct longitudinal trajectories of grip strength in both cohorts: accelerated decline and steady decline (Figure 2). In the HRS cohort, 30% of participants were classified into the accelerated decline group and 70% into the steady decline group. In the ELSA cohort, the corresponding proportions were 36.3 and 63.7%, respectively. The final two-group model showed acceptable model fit, with mean posterior probabilities exceeding 0.70 for all trajectory groups. Detailed model fit indices, including BIC, AIC, and log-likelihood values for competing models, are provided in Supplementary Table S1. Using the Steady decline group as the reference, participants in the Accelerated decline group exhibited a significantly higher risk of new-onset CVD after adjustment for potential confounders. The risk increased by 31%–44% (HRS: adjusted HR = 1.44, 95% CI: 1.22–1.70, *p* < 0.001; ELSA: adjusted HR = 1.31, 95% CI: 1.08–1.58, *p* = 0.005) (Table 4).

**Table 4:** FG regression analysis of the association between grip strength and its variation trajectory with the risk of new-onset CVD.

Exposure variable	Model 1		Model 2		Model 3		Model 4	
	sHR (95% CI)	*p*-value	sHR (95% CI)	*p*-value	sHR (95% CI)	*p*-value	sHR (95% CI)	*p*-value
HRS								
Grip per 1 SD	0.89 (0.84 ~ 0.94)	<0.001***	0.76 (0.70 ~ 0.83)	<0.001***	0.79 (0.73 ~ 0.86)	<0.001***	0.80 (0.73 ~ 0.87)	<0.001***
Grip trajectory								
Steady decline	1.00 (Reference)	—	1.00 (Reference)	—	1.00 (Reference)	—	1.00 (Reference)	—
Accelerated decline	1.17 (1.05 ~ 1.31)	0.005**	1.49 (1.27 ~ 1.75)	<0.001***	1.44 (1.22 ~ 1.68)	<0.001***	1.41 (1.20 ~ 1.66)	<0.001***
P for trend	0.91 (0.85 ~ 0.97)	0.005**	0.79 (0.72 ~ 0.87)	<0.001***	0.81 (0.73 ~ 0.89)	<0.001***	0.81 (0.74 ~ 0.90)	<0.001***
ELSA								
Grip per 1 SD	0.90 (0.85 ~ 0.95)	<0.001***	0.81 (0.74 ~ 0.89)	<0.001***	0.82 (0.75 ~ 0.90)	<0.001***	0.84 (0.76 ~ 0.92)	<0.001***
Grip trajectory								
Steady decline	1.00 (Reference)	—	1.00 (Reference)	—	1.00 (Reference)	—	1.00 (Reference)	—
Accelerated decline	1.09 (0.97 ~ 1.22)	0.160	1.32 (1.10 ~ 1.58)	0.002**	1.31 (1.10 ~ 1.57)	0.003**	1.29 (1.08 ~ 1.55)	0.005**
P for trend	1.00 (0.99 ~ 1.00)	0.160	0.99 (0.99 ~ 1.00)	0.002**	0.99 (0.99 ~ 1.00)	0.003**	0.99 (0.99 ~ 1.00)	0.005**

Model 1: crude.

Model 2: adjust: Age, Sex, Race, Education, marital status, Wealth, BMI.

Model 3: adjust: Age, Sex, Race, Education, marital status, Wealth, BMI, Hypertension, Diabetes, Drinking status, Smoking status.

Model 4: adjust: Age, Sex, Race, Education, marital status, Wealth, BMI, Hypertension, Diabetes, Drinking status, Smoking status, Physical activity, Cancer.

Significance: *p < 0.05, **p < 0.01, ***p < 0.001.

**Figure 3:**
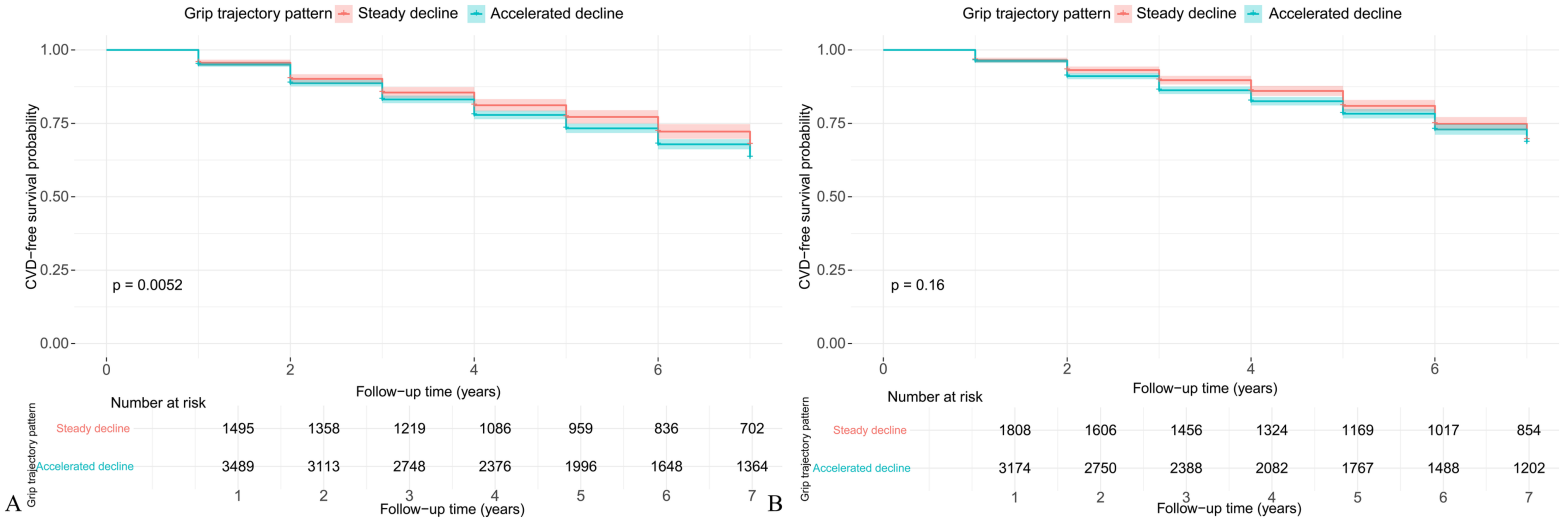
Kaplan–Meier curves for CVD-free survival according to grip strength trajectory groups: **(A)** HRS cohort; **(B)** ELSA cohort.

Kaplan–Meier survival curves demonstrated significant differences in CVD-free survival across the trajectory groups in the HRS cohort (log-rank test *p* < 0.0052). The divergence between the curves increased over time (Figure 3). In the ELSA cohort, the Steady decline group also showed a higher probability of CVD-free survival than the Accelerated decline group. However, this difference did not reach statistical significance (log-rank test *p* = 0.16) (Figure 3).

### 3.3 Competing risk analysis considering death as a competing event

Table 4 presents the results of the Fine–Gray competing risk regression analysis evaluating the association between handgrip strength, its trajectories, and new-onset CVD while treating death as a competing event. After accounting for the competing risk of death, the results remained consistent with those obtained from the Cox regression analysis.

After adjustment for potential covariates, higher handgrip strength was independently associated with a lower subdistribution hazard of new-onset CVD (HRS: adjusted sHR = 0.80, 95% CI: 0.73–0.87, *p* < 0.001; ELSA: adjusted sHR = 0.84, 95% CI: 0.76–0.92, *p* < 0.001). When participants were classified into Accelerated decline and Steady decline trajectory groups using GBTM, those in the Accelerated decline group exhibited a significantly higher sub distribution hazard of new-onset CVD compared with those in the Steady decline group. The risk increased by 29–41% (HRS: adjusted sHR = 1.41, 95% CI: 1.20–1.66, *p* < 0.001; ELSA: adjusted sHR = 1.29, 95% CI: 1.08–1.55, *p* = 0.005).

Trend tests based on the Fine–Gray model were also significant (HRS: adjusted sHR = 0.81, 95% CI: 0.74–0.90, *p* < 0.001; ELSA: adjusted sHR = 0.99, 95% CI: 0.99–1.00, *p* = 0.005). The magnitude and direction of these associations were comparable to those observed in the Cox regression analysis. This finding indicates that the relationship between handgrip strength, its trajectories, and new-onset CVD risk was not substantially affected by the competing risk of death (Table 4).

### 3.4 Discrete-time logistic regression analysis

Discrete-time logistic regression models were constructed using follow-up waves as the time unit. The dataset was reorganized into a person–period format. Both baseline handgrip strength and its trajectory patterns were significantly associated with the incidence of CVD during each follow-up interval.

In the fully adjusted model, handgrip strength remained inversely associated with new-onset CVD risk in both cohorts (HRS: adjusted OR = 0.78, 95% CI: 0.71–0.85, *p* < 0.001; ELSA: adjusted OR = 0.81, 95% CI: 0.73–0.89, *p* < 0.001). Participants in the Accelerated decline group had a higher incidence of CVD compared with those in the Steady decline group (HRS: adjusted OR = 1.47, 95% CI: 1.23–1.76, *p* < 0.001; ELSA: adjusted OR = 1.31, 95% CI: 1.07–1.60, *p* = 0.008).

The results from the discrete-time models were consistent in both direction and magnitude with those from the Cox regression and competing risk analyses, demonstrating the robustness of the findings across different time-modeling strategies (Table 5).

**Table 5:** Discrete-time logistic regression analysis of the association between grip strength and its variation trajectory and the risk of new-onset CVD.

Exposure variable	Model 1		Model 2		Model 3		Model 4	
	OR (95% CI)	*p*-value	OR (95% CI)	*p*-value	OR (95% CI)	*p*-value	OR (95% CI)	*p*-value
HRS								
Grip per 1 SD	0.88 (0.83 ~ 0.93)	<0.001***	0.74 (0.68 ~ 0.81)	<0.001***	0.77 (0.70 ~ 0.84)	<0.001***	0.78 (0.71 ~ 0.85)	<0.001***
Grip trajectory								
Steady decline	1.00 (Reference)	—	1.00 (Reference)	—	1.00 (Reference)	—	1.00 (Reference)	—
Accelerated decline	1.19 (1.05 ~ 1.34)	0.005**	1.56 (1.31 ~ 1.86)	<0.001***	1.50 (1.25 ~ 1.79)	<0.001***	1.47 (1.23 ~ 1.76)	<0.001***
P for trend	0.90 (0.84 ~ 0.97)	0.005**	0.77 (0.69 ~ 0.85)	<0.001***	0.79 (0.71 ~ 0.87)	<0.001***	0.79 (0.71 ~ 0.88)	<0.001***
ELSA								
Grip per 1 SD	0.89 (0.83 ~ 0.94)	<0.001***	0.79 (0.71 ~ 0.87)	<0.001***	0.80 (0.72 ~ 0.88)	<0.001***	0.81 (0.73 ~ 0.89)	<0.001***
Grip trajectory								
Steady decline	1.00 (Reference)	—	1.00 (Reference)	—	1.00 (Reference)	—	1.00 (Reference)	—
Accelerated decline	1.10 (0.97 ~ 1.25)	0.129	1.34 (1.10 ~ 1.63)	0.004**	1.34 (1.09 ~ 1.63)	0.005**	1.31 (1.07 ~ 1.60)	0.008**
P for trend	1.00 (0.99 ~ 1.00)	0.129	0.99 (0.99 ~ 1.00)	0.004**	0.99 (0.99 ~ 1.00)	0.005**	0.99 (0.99 ~ 1.00)	0.008**

Model 1: crude.

Model 2: adjust: Age, Sex, Race, Education, marital status, Wealth, BMI.

Model 3: adjust: Age, Sex, Race, Education, marital status, Wealth, BMI, Hypertension, Diabetes, Drinking status, Smoking status.

Model 4: adjust: Age, Sex, Race, Education, marital status, Wealth, BMI, Hypertension, Diabetes, Drinking status, Smoking status, Physical activity, Cancer.

Significance: *p < 0.05, **p < 0.01, ***p < 0.001.

### 3.5 Subgroup analysis

Subgroup analyses were conducted to assess whether the association between handgrip strength and new-onset CVD differed across population subgroups. Stratified analyses were performed according to sex, age, physical activity, and chronic conditions such as hypertension and diabetes.

**Figure 4:**
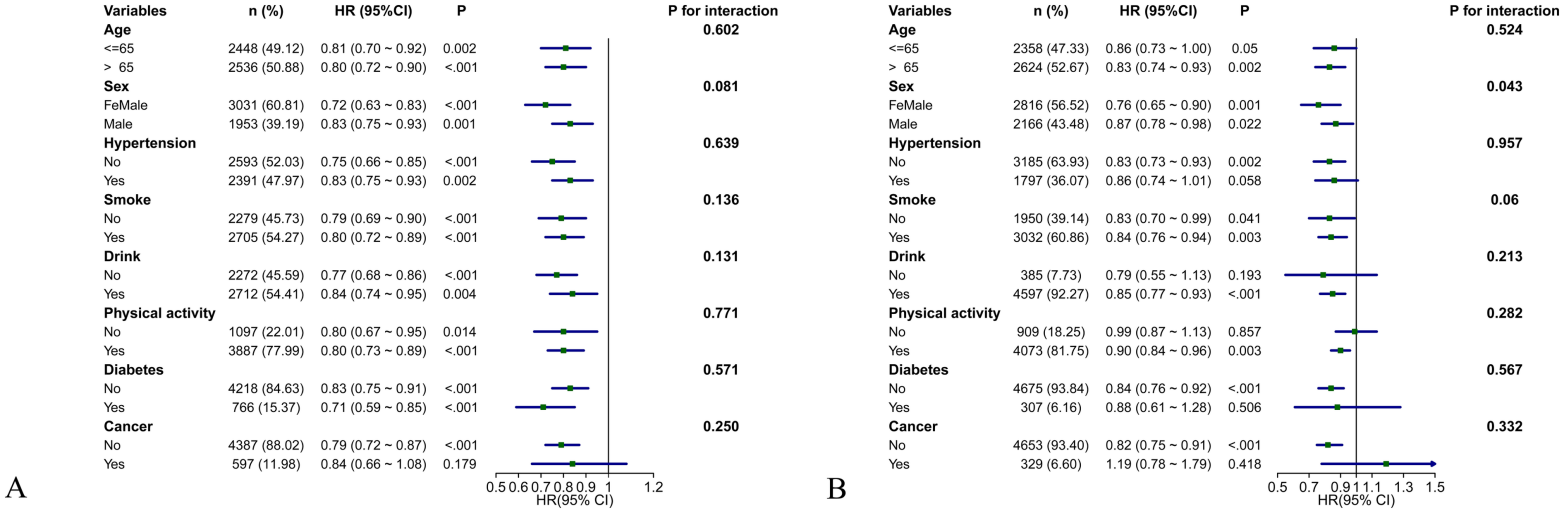
Forest plots of subgroup analyses showing the association between the grip strength and the risk of new-onset cardiovascular disease: **(A)** HRS cohort; **(B)** ELSA cohort.

The inverse association between handgrip strength and new-onset CVD showed minor variation across subgroups. In the HRS cohort, differences were observed according to cancer status. In the ELSA cohort, variations were observed across subgroups defined by hypertension, alcohol consumption, physical activity, diabetes, and cancer. However, interaction tests indicated that none of these subgroup variables significantly modified the association between handgrip strength and new-onset CVD risk (P for interaction > 0.05) (Figure 4).

### 3.6 Linear and nonlinear associations between handgrip strength and new-onset CVD

**Figure 5:**
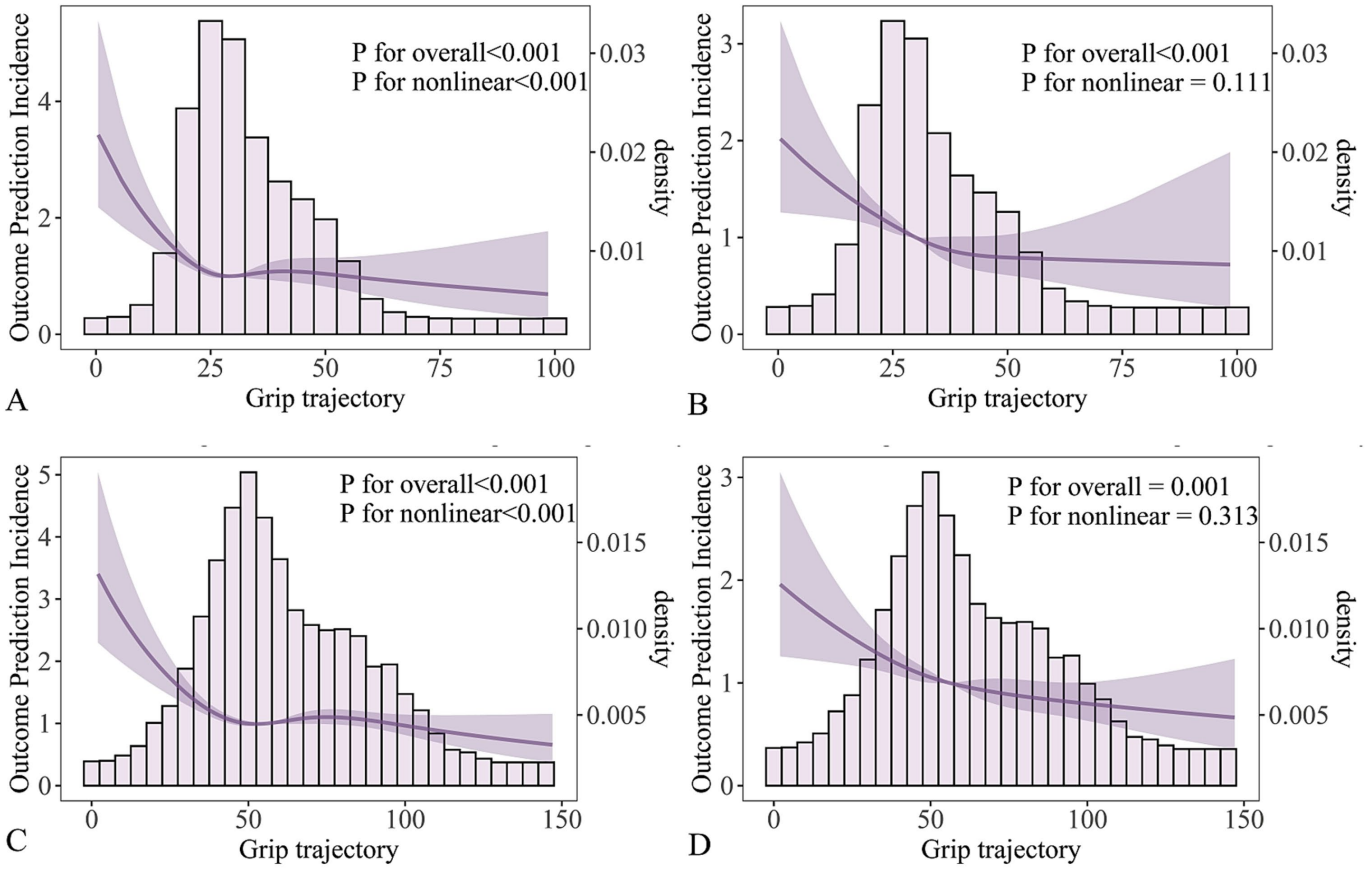
Restricted cubic spline analyses illustrating linear and nonlinear associations between the grip strength and the risk of new-onset cardiovascular disease: **(A)** unadjusted model, HRS cohort; **(B)** fully adjusted model, HRS cohort; **(C)** unadjusted model, ELSA cohort; **(D)** fully adjusted model, ELSA cohort.

RCS analysis was performed to examine the relationship between handgrip strength and new-onset CVD risk. The results are presented in Figure 5. In the unadjusted model, a nonlinear association between handgrip strength and new-onset CVD risk was observed in both the HRS and ELSA cohorts (*p* for nonlinear < 0.001 and p for overall < 0.001) (Figures 5A,C). After adjustment for potential confounders, the nonlinear relationship was no longer evident. Instead, a linear inverse association between handgrip strength and new-onset CVD risk was observed (HRS: p for nonlinear = 0.111 and p for overall < 0.001; ELSA: p for nonlinear = 0.313 and p for overall = 0.001) (Figures 5B,D).

### 3.7 Mediation analysis

**Figure 6:**
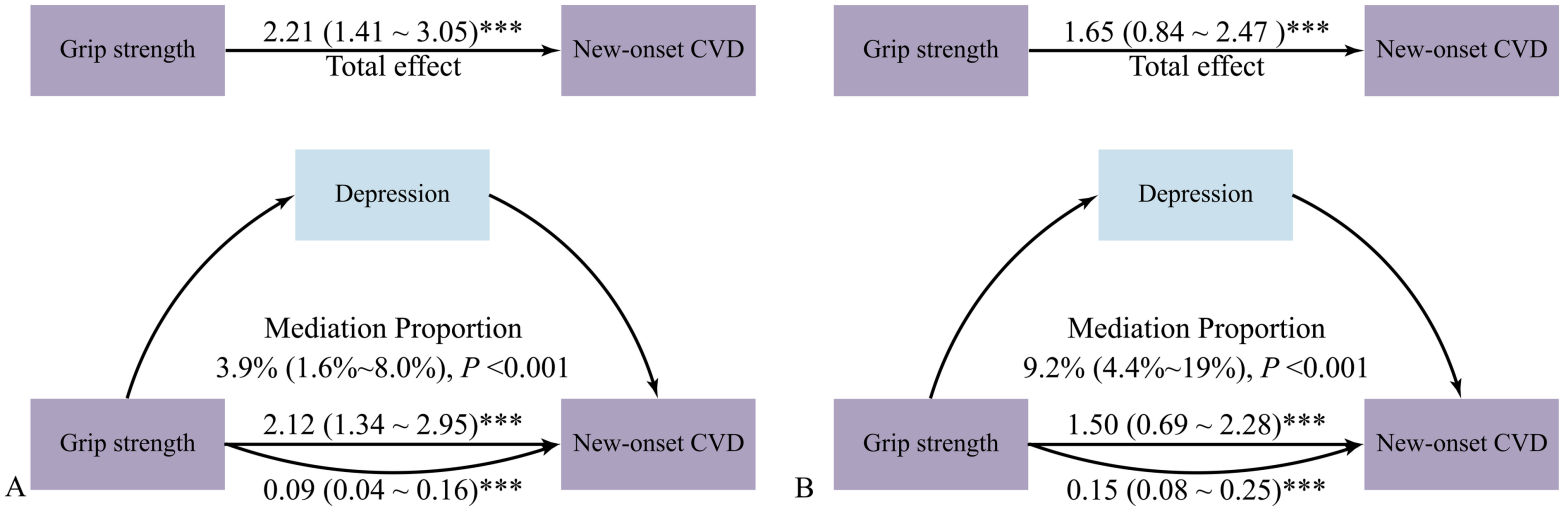
Mediation analysis demonstrates the role of depression in the relationship between baseline grip strength and the risk of new-onset CVD: **(A)** HRS cohort; **(B)** ELSA cohort.

Figure 6 illustrates the potential mediating role of depression in the association between baseline handgrip strength and new-onset CVD risk. Depression partially mediated this association in both cohorts. In the HRS cohort, the mediating effect accounted for approximately 3.9% of the total association, whereas in the ELSA cohort, the corresponding proportion was approximately 9.2%. Both the direct association and the total association remained statistically significant in the two cohorts.

## 4 Discussion

This study systematically examined the associations between handgrip strength, its long-term trajectories, and the risk of new-onset CVD using two nationally representative longitudinal aging cohorts from the United States and the United Kingdom. The results indicate that higher baseline handgrip strength is significantly associated with a lower risk of new-onset CVD. This association remained robust after comprehensive adjustment for potential confounders, including sociodemographic characteristics, lifestyle behaviors, and chronic health conditions. Trajectory analysis further revealed heterogeneous patterns of handgrip strength change across individuals. Participants who experienced an accelerated decline in handgrip strength had a substantially higher risk of developing CVD compared with those whose grip strength declined more gradually. These findings were consistent across competing risk analyses and discrete-time models, supporting the robustness of the observed associations. In addition, mediation analysis suggested that depression partially mediated the relationship between handgrip strength and CVD risk, indicating that both physical functional decline and psychological health may contribute to the development of cardiovascular disease.

Previous studies have consistently reported that lower baseline handgrip strength is associated with increased risks of cardiovascular events, cardiovascular mortality, and all-cause mortality. As a result, handgrip strength has been proposed as a potential marker of frailty or biological aging (20–22). However, most previous investigations relied on a single baseline measurement and treated handgrip strength as a static exposure. Such an approach cannot capture the progressive and heterogeneous decline in muscle function that occurs during biological aging (23). More recent research has therefore focused on longitudinal changes in physical function indicators, which may provide more sensitive predictors of chronic disease risk (24, 25). Despite this shift in research focus, evidence directly linking trajectories or rates of decline in handgrip strength to subsequent cardiovascular disease incidence remains limited. Existing studies have also been largely restricted to single-cohort analyses (16, 26). By incorporating repeated assessments of handgrip strength and applying group-based trajectory modeling in two national cohorts, the present study extends the existing literature. The findings demonstrate that both the rate of decline and distinct trajectory patterns provide additional prognostic information beyond baseline grip strength levels.

The biological and behavioral mechanisms linking accelerated decline in handgrip strength to elevated cardiovascular risk are complex but supported by substantial evidence. Handgrip strength can serve as a surrogate indicator of overall skeletal muscle mass and functional capacity. Rapid declines in muscle strength are closely associated with chronic low-grade inflammation, characterized by elevated circulating levels of interleukin-6, tumor necrosis factor-*α*, and C-reactive protein. These inflammatory processes can promote endothelial dysfunction, increase arterial stiffness, and accelerate the development of atherosclerosis (27, 28). In addition, reduced muscle strength is strongly associated with insulin resistance and metabolic syndrome. These metabolic disturbances impair glucose uptake in skeletal muscle and contribute to vascular injury through mechanisms involving hyperinsulinemia and dyslipidemia (29, 30). From a broader aging perspective, accelerated loss of handgrip strength may reflect systemic biological aging processes, including mitochondrial dysfunction, oxidative stress, and autonomic imbalance. Each of these factors independently contributes to increased cardiovascular vulnerability (31, 32). Behavioral pathways may also play an important role. Individuals experiencing rapid declines in muscle function often exhibit lower levels of physical activity, poorer nutritional status, and increasing frailty. These conditions may further elevate cardiovascular risk by reducing cardiopulmonary reserve and increasing susceptibility to physiological stressors (4, 6). In addition, the mediation analysis suggested that depression may partially explain the association between lower handgrip strength and increased new-onset CVD risk. Older adults with reduced muscle strength may be more susceptible to depressive symptoms due to functional limitation, reduced social participation, and declining quality of life. Depression, in turn, has been associated with chronic inflammation, autonomic dysregulation, and adverse cardiovascular behaviors, which may contribute to increased cardiovascular risk. However, these mediation findings should be interpreted cautiously because the observational nature of the study precludes definitive causal inference.

These findings have important clinical and public health implications. Measurement of handgrip strength is inexpensive, rapid, and highly reproducible, requiring only a handheld dynamometer. Consequently, it is well suited for routine implementation in community screening programs, primary healthcare settings, and geriatric clinics worldwide (33, 34). Longitudinal monitoring of changes in handgrip strength may complement, or even precede, traditional CVD risk prediction tools. Functional decline may become detectable earlier than alterations in conventional biomarkers, thereby enabling earlier identification of high-risk individuals and facilitating timely interventions. Such interventions may include resistance training programs or nutritional optimization aimed at preserving muscle function and reducing the incidence of cardiovascular events (18, 35). In the context of global population aging, this simple and dynamic functional indicator provides a scalable strategy for risk stratification and primary prevention, with the potential to substantially reduce the societal burden of cardiovascular disease (19).

Several design features were implemented to reduce potential reverse causation. Specifically, participants with existing CVD during the trajectory assessment period were excluded, and handgrip strength trajectories were derived from repeated measurements collected before outcome follow-up. These procedures strengthened the temporal relationship between exposure and subsequent new-onset CVD risk.

This study has several notable strengths. First, it utilized two large, nationally representative longitudinal cohorts with extended follow-up periods. Second, repeated measurements of handgrip strength enabled the construction of detailed trajectory models, allowing a more comprehensive evaluation of dynamic changes in muscle function. Third, extensive adjustment for potential confounders was performed, and multiple sensitivity analyses, including competing risk models and mediation analyses, were conducted to ensure the robustness of the findings. Finally, the consistency of results across two cohorts from different healthcare systems and sociocultural contexts strengthens both the internal validity and the external generalizability of the study (7, 9).

Nevertheless, several limitations should be considered. CVD outcomes were based on self-reported physician diagnoses rather than adjudicated clinical records, which may have introduced recall bias or outcome misclassification. In addition, the specific subtype and severity of cardiovascular conditions could not be fully distinguished within the available survey data. However, validation studies in both the HRS and ELSA cohorts have demonstrated good agreement between self-reported diagnoses and medical records (13). Residual confounding from unmeasured or incompletely measured factors cannot be completely excluded. Although extensive covariate adjustment was performed, important variables such as dietary and nutritional status, medication use, inflammatory biomarkers, and sarcopenia or frailty-related indicators were not comprehensively available or harmonized across the two cohorts. These factors may potentially influence both handgrip strength and cardiovascular risk. In addition, the study population consisted primarily of middle-aged and older adults; therefore, the applicability of these findings to younger populations requires further investigation. Finally, the mediating effect of depression was relatively modest, suggesting that additional biological or behavioral pathways may also contribute to the association between handgrip strength decline and cardiovascular disease risk and warrant further exploration (14).

## 5 Conclusion

In summary, this study demonstrates that both lower baseline handgrip strength and accelerated declines in handgrip strength trajectories are significantly associated with an increased risk of new-onset CVD among older adults. Compared with a single measurement of handgrip strength, dynamic assessment of longitudinal changes provides a more comprehensive representation of long-term muscle function and may offer additional value for cardiovascular risk evaluation. Routine monitoring of handgrip strength changes may therefore have important clinical and public health implications for the health management of aging populations. Future research should further investigate the biological mechanisms linking muscle functional decline to cardiovascular disease and evaluate whether interventions aimed at improving muscle function can reduce the risk of cardiovascular events.

## Data Availability

The original contributions presented in the study are included in the article/Supplementary material, further inquiries can be directed to the corresponding authors.
