# Supplementary material for: Trajectories of grip strength decline and risk of new-onset cardiovascular disease: evidence from the HRS and ELSA cohorts
Source: Front Public Health. 2026 Jun 26;14:1836439. doi: 10.3389/fpubh.2026.1836439 (PMC13352478; doi:10.3389/fpubh.2026.1836439)
Supplement: Supplementary file 2 [file Table_1.docx]

# Supplementary Table 1. Model fit statistics for competing trajectory models in HRS and ELSA cohorts

| **Model** | **Polynomial Order** | | **AIC** | | **BIC** | **Log-Likelihood** | **Smallest Group (%)** | **Mean Posterior Probability** |
| --- | --- | --- | --- | --- | --- | --- | --- | --- |
| HRS |  | |  | |  |  |  |  |
| 1-class | Linear | | 84324.6 | | 84363.8 | -42156.3 | 100.0 |  |
| 2-class | Linear | | 83266.8 | | 83404.5 | -41612.4 | 41.2 | 0.82 |
| 3-class | Linear | | 83587.0 | | 83658.4 | -41782.5 | 4.7 | 0.86 |
| 4-class | Linear | | 83412.2 | | 83516.7 | 41690.1 | 2.1 | 0.89 |
| Elsa |  | |  | |  |  |  |  |
| 1-class | Linear | | 79781.4 | | 79819.6 | -39884.7 | 100.0 |  |
| 2-class | Linear | | 78678.4 | | 78812.5 | -39318.2 | 36.9 | 0.81 |
| 3-class | Linear | | 79006.6 | | 79076.1 | -39492.3 | 3.8 | 0.85 |
| 4-class | Linear | | 78823.0 | | 78924.8 | -39395.5 | 1.9 | 0.88 |
|  | |  | |  |  |  |  |  |
